# Validation of band counts in eyestalks for the determination of age of Antarctic krill, *Euphausia superba*

**DOI:** 10.1371/journal.pone.0171773

**Published:** 2017-02-22

**Authors:** Raouf Kilada, Christian S. Reiss, So Kawaguchi, Robert A. King, Tsuyoshi Matsuda, Taro Ichii

**Affiliations:** 1 University of New Brunswick, Saint John, Canada and Department of Marine Science, University of Suez Canal, Ismailia, Egypt; 2 NOAA Fisheries, Antarctic Ecosystem Research Division, La Jolla, California, United States of America; 3 Australian Antarctic Division, Kingston, Tasmania, Australia; 4 Antarctic Climate and Ecosystems Cooperative Research Centre, Hobart, Tasmania, Australia; 5 Port of Nagoya Public Aquarium, Nagoya, Japan; 6 National Research Institute of Far Seas Fisheries, Yokohama, Japan; University of Shiga Prefecture, JAPAN

## Abstract

Using known-age Antarctic krill (*Euphausia superba*) grown from eggs hatched at two different laboratories, we validate the annual pattern of bands deposited in the eyestalks of krill and determine the absolute age of these animals. Ages two through five years were validated, and these animals ranged from 37.1 to 62.6 mm in total length. The band counts in these individuals were either identical to their absolute ages, or only failed to agree by a few months, which demonstrates the accuracy of this method. Precision and bias were estimated graphically using Chang’s index (Coefficient of Variation = 5.03%). High accuracy and precision between readers and low ageing bias indicate that longitudinal sections of eyestalks can be used to age krill in wild samples and to develop age-based stock assessment models for krill. Archival samples preserved in formalin (5%) and stored in ambient conditions were also readable. Ageing preserved krill will provide the opportunity to examine changes in growth among krill populations within the Southern Ocean and to retrospectively examine changes in krill production over the last century to better understand the historical and future impacts of climate change on this critical Southern Ocean species.

## Introduction

Euphausiids are a critical link in the pelagic food webs of many marine ecosystems [1]. Several species dominate the pelagic communities in oceans world-wide, and their life-history plasticity is an important aspect of their success [2]. Euphausiids are a main component in structuring the energy flow through marine ecosystems, and these species respond to environmental variability through their growth rates, fecundities, and dietary flexibility. In the Southern Ocean, Antarctic krill (*Euphausia superba*; hereafter “krill”) is a key ecological species south of the Antarctic Polar Front [3]. It has an estimated biomass in excess of 2 x 10^8^ t [4, 5], with one-quarter of this biomass concentrated in just 10% of its total habitat area, specifically the Bellingshausen and Scotia Seas. Additionally, this region supports a high biomass of predators, including fishes, seabirds, and marine mammals that consume approximately 48 million tonnes of krill annually [6]. Krill are also the basis of a significant and growing commercial fishery [7] that is currently under-exploited, but with future development could equal 7% of current global marine capture fisheries production [8].

The krill fishery in the Southern Ocean is managed by the Commission for the Conservation of Antarctic Marine Living Resources (CCAMLR). CCAMLR is developing a spatially-structured management approach to limit the impacts of fishing on the krill stock and krill-dependent predators [9], and will rely on biomass surveys of krill populations using both echo-sounders and net tows to estimate the size of these populations [10]. The results of these surveys will then be used to set catch limits for the fishery [11]. At present, however, age-based stock assessment models are unavailable and CCAMLR uses data from length-frequency distributions to infer growth rate and length-at-age for input into the General Yield Model that is used to determine the theoretical catch limit. Unfortunately, these length-based methods are inherently imprecise due to the plasticity of krill life history (e.g., [12]).

For euphausiids, an additional difficulty with length-based assessment methods is that animals can ‘shrink’, either through changes in size during the regular molt, during periods of seasonal dormancy or food limitation, or during periods of intensive reproduction (e.g., [13, 14, 15, 16, 17, 18]). Thus, it has remained difficult to compare spatial and temporal variability in growth rates using length-frequency distributions because it is not possible to separate fast-growing young krill from slow-growing old krill. This limitation has meant that despite more than 50 years of research, it has not been possible to accurately assess the age structure of krill populations or to estimate their natural longevity [19, 20]. This limitation has also hampered comparisons of length-at-age among areas of the Southern Ocean with different environmental conditions [21, 22]. Given the magnitude of the climate-driven changes in the Southern Ocean, and the regional differences in such changes (e.g., with faster loss of sea ice in the Antarctic Peninsula region) determining the variability in growth among areas or over time is critical to understanding the response of krill to future climate change [23, 20, 24].

Unlike fishes and invertebrates that record their age and growth in annuli deposited and preserved in calcified structures like otoliths, scales, and shells, krill do not have hard parts that are preserved during ecdysis [25]. Also, unlike birds and mammals that can be tagged at their breeding areas, krill are too small and numerous to use tagging data to estimate growth, mortality, and age. The lack of hard parts has resulted in considerable effort to develop indirect methods to determine age and growth in order to augment or replace length-based estimates of age. Among the methods used to estimate age in crustaceans has been the measurement of accumulated auto-fluorescing lipofuscin pigments in the brain or eyestalk neural tissue. This metabolic by-product provides an index of physiological (i.e., metabolic-dependent) age [26, 27, 28, 29]. With this method, cohorts are identified by modal analysis of lipofuscin concentrations in population samples, or individual ages are ascribed based on lipofuscin accumulation in tagged or laboratory animals. However, the rate at which lipofuscin is accumulated can be dependent on temperature, diet, and other factors [30], complicating the determination of age. Other indirect measures of krill age and growth have been developed using structures that are not resorbed during ecdysis. These include the number of cones in the compound eye and the size of the eyeball [31, 32]. These studies showed that these structures can be used to examine the response of krill growth to recent environmental conditions. However, like the biochemical methods described above, environmental influences (food and temperature) impact krill growth and therefore the number of cones and the size of the eyeball. These techniques do not necessarily provide a more robust estimate of krill age compared to length-based methods [20].

Recently, Kilada et al. [33] showed that annual growth bands are formed in the eyestalk of different shrimp species. The authors corroborated the annual formation of these marks using other methods, including marking and tagging [25], and concluded that these growth bands indicate absolute age. Since this original work, further studies on a number species have been conducted [33, 34, 35, 36, 37]. In krill, Reiss et al. [38] first documented growth bands in thin sections of the eyestalk. Krafft et al. [39] further examined the potential use of longitudinal sections of eyestalk in relation to sex and length in krill, but did not validate the method.

Here, we validate for the first time, the direct determination of absolute age in krill using bands visible in thin section of eyestalks of known-age krill collected opportunistically from two different laboratory facilities: the Australian Antarctic Division (AAD) and the Port of Nagoya Public Aquarium (PNPA). We also show that age bands are visible in historical formalin-preserved samples collected from the wild. The implications of our research are substantial because our results demonstrate that direct comparisons of length-at-age can be made between krill collected in different areas of the Southern Ocean and that age-based assessment models can be developed for krill. Additionally, because we show that archival samples can be aged, retrospective studies of krill growth between and among areas over time are possible, allowing for the assessment of how climate and ecosystem change affect krill populations in the Southern Ocean.

## Methods and materials

### Known-age samples

Krill of known age were grown from eggs at the Australian Antarctic Division (AAD) in Australia [40] and the Port of Nagoya Public Aquarium (PNPA) in Japan [41, 42], the only two institutions that are presently capable of reproducing krill in captivity. At both institutions, large groups of krill (200–2000 individuals) were kept in large holding tanks controlled at either 0.5°C (AAD) or 1.0°C (PNPA) and fertilized naturally. Gravid females bearing spermatophores were individually separated into spawning jars to harvest embryos, which were raised to adulthood in known-age groups in larger tanks. Details of the facility setups and general feeding regimes are described in Kawaguchi et al. [40] for AAD and in Hirano and Matsuda [41, 42] for PNPA. The experimental setup of the seasonal light regimes differed between the two facilities. At the AAD, a sinusoidal annual cycle with monthly variations in the photoperiod and daily variation in light intensity was used. The light regime approximated the same cycle that is found at 66°S and 30 m depth. Specifically, this regime used a maximum light intensity of 100 lux at the surface of the tank (approximating the 1% light level at 30 m) during summer (December) at midday, and declined until animals were kept in complete darkness throughout the day during mid-winter (June). At the PNPA, the light intensity of the aquaria was held constant at 120 lux throughout the year, and only light period varied. Light period was set for 18 hours in December, and was reduced stepwise by two hours every month (i.e., 16 hours in January and November, 14 hours in February and October, 12 hours in March and September, 10 hours in April and August, 8 hours in May and July, and 6 hours in June).

Krill from AAD were preserved fresh in an ethanol, water, glycerol (70:26:4 by volume) preservative, whereas krill from PNPA were preserved in formalin when dead individuals were found in the tank during daily inspections. Therefore, at PNPA, krill were preserved up to 24 hours after death.

### Field collections

Krill age was determined from individuals collected from two sample sets: summer (January and February of 1992–2005) and winter (August 2015). The former set was collected during annual U.S. Antarctic Marine Living Resources (AMLR) Program krill surveys around the South Shetland Islands using a 2.54 m^2^ mouth area plankton trawl [43]. All samples were collected in accordance with CCAMLR Conservation Measure 24–01. This study did not sample endangered or protected species. Krill were preserved immediately after capture in buffered formalin (5%) and stored in 1 liter glass jars at ambient temperatures until 2009, when samples were moved to a climate-controlled indoor facility. For this study, a total of 87 krill were randomly sampled from the summer samples. However, of these 87 animals, 37 were damaged during the initial preparation for ageing, leaving 50 individuals for embedding and sectioning. Of the 50 useable animals, 25 were used for training technicians on the technique to embed and section the eye stalk (see below), and we attempted to estimate ages from the remaining 25.

Krill were also collected in the austral winter of 2015 from around the South Shetland Islands using the same gear and in the same general areas as the archival samples. A total of 73 individuals were randomly selected from stations and immediately preserved in the glycerol preservative. Krill were placed individually in cuvettes, and then stored indoors at 20°C. Eighteen of these samples were used for training, and of the remaining samples, 40 were readable after sectioning.

### Sample processing

**Fig 1:**
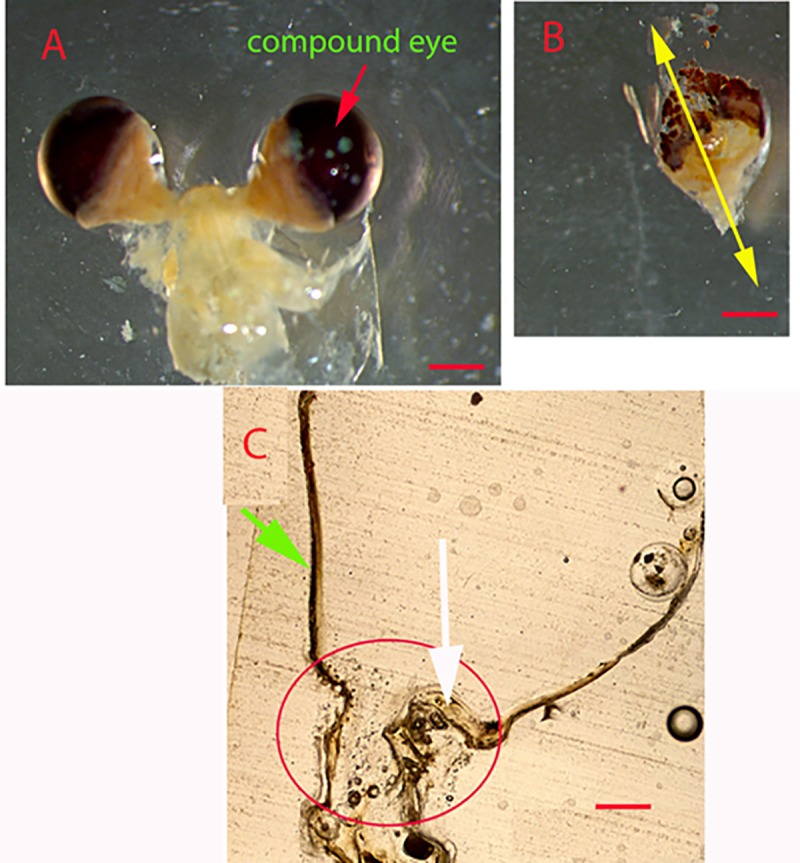
Eyestalk of Antarctic krill. (A) Eyestalk pair before cleaning; (B) cleaned left eyestalk, with yellow arrows indicating the cutting axis; and (c) thin section of the whole eyestalk showing the growing edge or the epicuticle (green arrow), the endocuticle (white arrow), and the location that shows clear growth bands (red circle). Scale bar indicates 200 μm.

**Fig 2:**
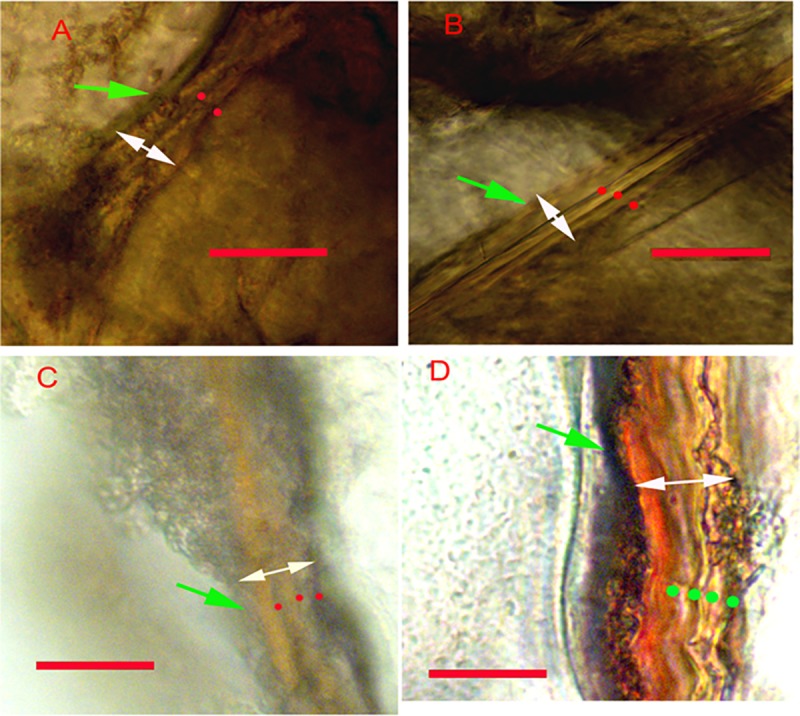
Thin sections of known-age Antarctic krill eyestalks. Transverse thin sections (170 μm) in the eyestalk of known-age individuals of Antarctic krill showing the annual growth bands indicated by dots. Dots are colored green in Figure D for contrast. Green and white arrows indicate the epicuticle and endocuticle layers, respectively. Scale bars indicate 20 μm. A: 37.8 mm total length (TL) and 1 year (y) 4 month (m) old; B: 41.1 mm TL and 3 y 5 m old; C: 43.7 mm TL and 3 y 5 m old; D: 52.2 mm TL and 4 y old.

Eyestalks were obtained by dissecting preserved krill (Fig 1A), removing the compound eye and all tissues filling the eyestalk (Fig 1B). After leaving the clean structure for 2–3 minutes until dry, the structures were embedded flat in Cold Cure epoxy resin on the bottom of silicone wells. The embedded structure was left for 48 hours until the epoxy resin hardened. The resin block was cut along a longitudinal axis (Fig 1B) to prepare serial longitudinal sections (160–180 μm thickness) with a diamond-bladed Isomet saw (see S1 Fig for schematic). The product of this step is a funnel-shaped section where the outer layer is the exocuticle as shown in Fig 1C and Fig 2. Sections were polished by hand using dry 0.3 μm grit lapping film, and viewed with transmitted light under 90% ethyl alcohol with a CX41 Olympus compound microscope under 4 to 10 x magnification. Digital images were taken with a DP72 Olympus video camera attached to the microscope, and images were digitally enhanced, if necessary, using Adobe Photoshop 12.0.4 to increase the contrast between adjacent bands. Growth bands can be recognized as paired light and dark zones in the endocuticle. Annuli were counted from the basal (adjacent to the membranous layer and hypodermis) to the distal region of the endocuticle without knowledge of the animals’ actual age or length.

As noted above, not all krill selected and mounted in epoxy were useable. In some cases, krill preserved in formalin were brittle and fractured when cleaned or sectioned. In other cases, sections were not useable because of the angle of the cuts. We did not quantify the reasons for exclusion of each sample, but note that with experience the proportion of samples that were useable increased.

### Precision assessment

To examine precision, band counts from a random selection of mounted eyestalk samples from winter and summer collections (above) were made independently by two readers for each specimen without prior knowledge of the krill length or of previous counts. Age-determination bias between readers was assessed through the use of an age-bias plot (S1 File). This type of graph displays band counts of one reader against a second reader in reference to an equivalence line where Reader 1 has the same results as Reader 2. Specifically, for all animals assigned a given age by Reader 1, the mean age and 95% confidence intervals of the ages assigned by Reader 2 are plotted against the age determined by Reader 1 (25). Precision estimates were calculated by using the coefficient of variation (CV) as described by Chang (44) as follows:

$${CV}_{j}=100*\frac{\sqrt{\sum_{i=1}^{R}{\left(X_{ij}-\bar{x}\right)}^{2}}}{{\bar{x}}_{j}}$$

where *X*_*ij*_ is the *i*^*th*^ age estimate of the *j*^*th*^ krill, $\bar{x}$ is the mean age of the *j*^*th*^ krill, and *R* is the number of times each krill is aged. The CV is averaged across krill samples of a given age to produce a mean CV.

## Results and discussion

### Age-validation and reader bias

Of the 19 original known-age krill in this study, 11 were successfully aged (six from AAD and five from PNPA). Krill from AAD ranged from 37.2 to 42.1 mm and were between 1.4 and four years of age. Krill from PNPA ranged from 36.2 to 62.6 mm and were between three and five years of age (Table 1). Differences between band counts and actual ages were always within a few months of, or identical to, the known age. For example, two bands were present on a 37.8 mm krill that was 1.4 years old (Fig 2A). In this case the second band was not fully deposited, yet it was visibly wide enough to consider it as the second band. In a 52.2 mm krill that was four years old, four bands were counted. This difference is explained by our inability to estimate ages to the day or month. These results indicate that band counts are annual marks and can be used as an absolute age indicator.

**Table 1:** Information on known-age krill used in age validation. AAD–Australian Antarctic Division; PNPA–Port of Nagoya Public Aquarium. Estimated absolute age in years (y) and months (m). Plusses (+) and minuses (-) represent the relative difference in the age given the hatch year, and the date of death assuming a January 1 birthdate.

ID	TL (mm)	Hatching Date	Sampling Date	Absolute Age (years, months)	Bands counted
AAD #1	43.7	Sept. 2011	Feb. 2015	3y, 5 m	3
AAD #2	41.1	Sept. 2011	Feb. 2015	3y, 5 m	3
AAD #1003	52.2	2011	Sept. 2015	4y	4
AAD#7	37.8	Oct. 2013	Feb. 2015	1y, 4 m	2
AAD#8	42.8	Oct. 2013	Feb. 2015	1y, 4 m	2
PNPA #1	43.0	2006–07	March 26, 2010	4+ y	4
PNPA #2	36.2	2006–07	March 26, 2010	4+ y	4
PNPA #5	62.6	2006	Dec. 5, 2011	5y	5
PNPA #6	52.0	2012	Nov. 25, 2015	3y	3
PNPA #7	55.4	2011	Sept. 11, 2015	4- y	4
PNPA #8	52.2	2011	Sept. 14, 2015	4- y	4

**Fig 3:**
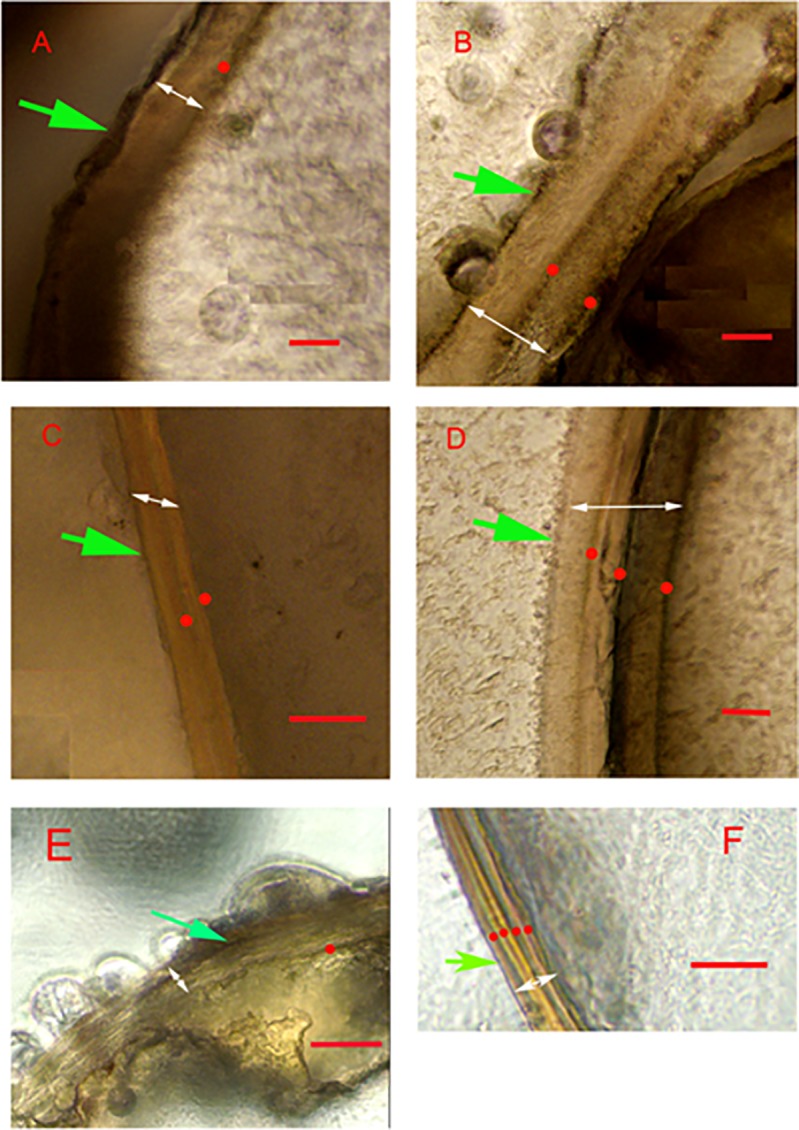
Thin sections of Antarctic krill eyestalks collected from the wild. Transverse thin sections (170 μm) in the eyestalk of individuals of Antarctic krill collected from the wild in summer 1992. (A: 33 mm TL; B: 47 mm TL; C: 46 mm TL; D: 48 mm TL) and samples collected in 2015 (E: 29 mm TL; F: 45 mm TL). The images show the annual growth bands indicated by dots. Green and white arrows indicate the epicuticle and endocuticle layers, respectively. Scale bars indicate 20 μm.

**Fig 4:**
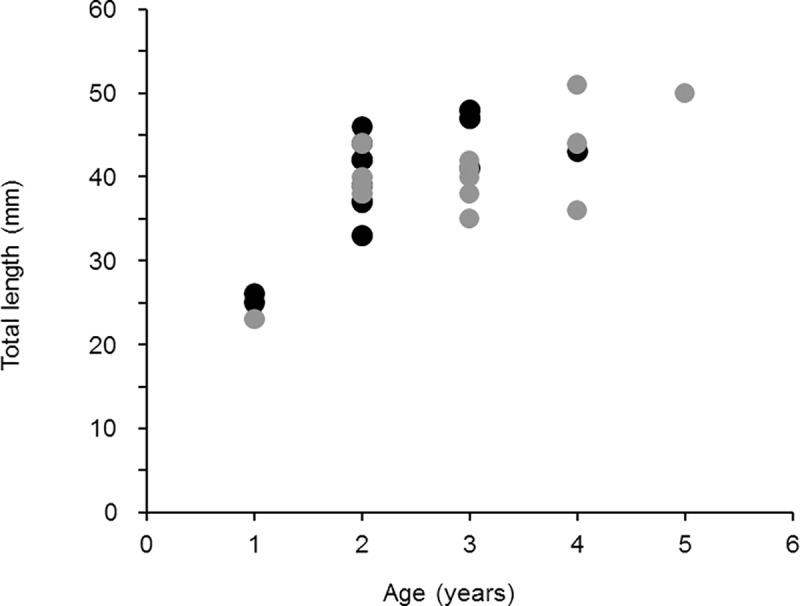
Scatterplot of variability in lengths at age for Antarctic krill. Krill lengths varied widely with age, beginning at age two. Formalin preserved summer samples are represented by the black symbols while winter samples preserved in glycerol are represented by the grey symbols.

Twenty-nine krill were successfully aged from formalin- and glycerol-preserved wild krill. Although they were more difficult to dissect and process, formalin-preserved samples were readable, and growth bands were visible in thin sections (Fig 3A–3D). Band counts from formalin samples ranged from one to four over a length range of 25 to 48 mm. Samples preserved in the glycerol preservative were easier to prepare (more pliable and less fragile), and bands were clearly visible in these samples. Band counts of these samples also ranged from one to five over a length range of 23 to 51 mm (Fig 4).

**Fig 5:**
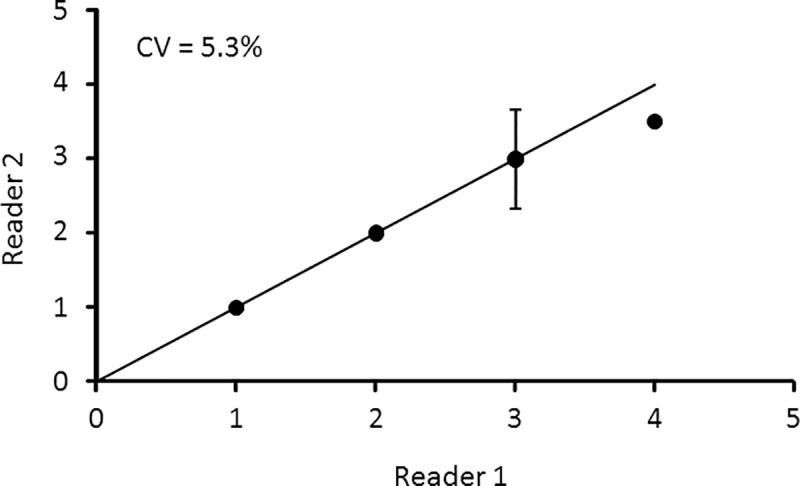
Bias plot of Antarctic krill band count readings. Age bias graph for Antarctic krill aged by Readers 1 and 2 that counted the growth bands in thin sections of eyestalk. Each error bar represents the 95% confidence interval about the mean age assigned by Reader 2 to all krill individuals assigned a given age by Reader 1. The values indicate the number of individuals aged at each age group. The solid line represents 1:1 equivalence.

Reader bias of ageing unknown-age krill from the formalin- and glycerol-preserved samples was assessed by comparing band counts from 20 randomly-picked prepared samples. Band counts were consistent between two independent readers (Fig 5). The precision of the readers was high, with a coefficient of variation (CV) of 5.2%.

Thin sections of the eyestalks of krill revealed distinct bands within the endocuticle that were consistent in number with known ages of animals hatched and raised to adulthood in captivity. As with other crustacean Families [33], all three layers of the cuticle–the epicuticle, exocuticle, and endocuticle, as ordered from the external surface—were visible in thin sections of the eyestalks of individual krill.

The bipartite growth bands in all eyestalk sections consisted of a broad translucent zone bordered by a narrower dark band (Fig 2). These bands were wider than the laminae that were occasionally observed within areas of the endocuticle. Although cuticle material is shed during ecdysis, it is apparent from chemical marking that some mineralized features of the cuticle are retained post-ecdysis. Kilada et al. [33] demonstrated that chemical marks were retained in the eyestalk of the American lobster (*Homarus americanus*) after three moults. Further, Leland et al. [45] validated the annual deposition of growth bands on the gastric mill ossicles of the freshwater crayfish (redclaw, *Cherax quadricarinatus*) using chemical tagging. Kilada et al. [33] suggested that mineralized features of certain parts of the cuticle are retained, and retention of this material may explain how crustaceans retain growth bands over time. The seasonal cycle in light used in the experiments may have provided a sufficient zeitgeber to generate the annual banding observed in animals from both the AAD and PNPA. While further research is required to clarify the physiological mechanism by which euphausiids deposit and retain the bands and some questions remain [46, 47], the banding pattern observed in krill is consistent with the broader pattern that seems robust across decapod crustaceans.

The use of two different sources of known-age animals for age validation is an important factor that gives us confidence that the bands are annual and are direct measures of age. Krill moult with a frequency that is strongly dependent on water temperature and do not have a terminal moult [48]. Given the different environmental conditions at the AAD and PNPA aquaria, moulting frequency would be expected to differ. Had moulting frequency significantly affected the deposition of band material in the endocuticle, consistent annual band counts between animals from different aquaria would not be expected. The consistent counts across laboratories support the hypothesis that band deposition is independent of moulting frequency, and that bands are deposited once per year.

Krill are spawned between November and February in many areas of the Antarctic, and are therefore one year of age the following January (when surveys are often conducted during the austral summer). This fixes the “birth date” to January. For winter samples, 1-year-old krill are 8 months older, but because krill can shrink through ecdysis during winter [15, 48], complications in interpreting the annular bands may arise. In this study, we examined 1-year-old krill that were collected in summer (January to March) and winter (August) and therefore differed in age by 8 months. Because we are uncertain when the first annular mark is deposited, significant ageing errors could arise when comparing lengths at age of the youngest krill.

Despite the differences in age for winter and summer samples, when combined, the estimate of reader bias was relatively low and consistent with other studies [39]. It was also consistent with established estimates for most fishes (5%–12%; [2]) and bivalves (5%–7%; [49]). The CV was also similar to other shrimp species, including northern shrimp (*Pandalus borealis*) (8%; [33]) and Chilean Nylon shrimp (*Heterocarpus reedi*) (7.2%; [34]). The low bias and low CV in this study suggests that proper training of readers, coupled with cross-training and use of reference samples, should enable high-quality age estimates for krill.

Proper determination of lengths-at-age and estimation of growth rates sufficient for spatio-temporal comparisons will require further research to ensure that biases will not influence estimates [e.g., 50]. Recent work on the modeling of age and growth has reiterated the possible ways that biases can enter into the estimation procedure and highlighted that these biases can be substantial. Spatial differences in growth, population mixing, incomplete sampling of the age structure, and oversampling for older animals need to be carefully assessed. Accounting for these sources of bias, a priori, and modeling length-at-age using modern techniques offer the best opportunity to develop robust comparisons of length-at-age among years from preserved samples collected with different sampling gears.

A number of studies are needed to continue the development of age validation of the longitudinal sections of eyestalks for age determination in krill and other euphausiids. Repeating this opportunistic experiment with larger numbers of krill would be beneficial, but will take a number of years. Additionally, it would be useful to examine the relationship between band counts and other measures of size that have been proposed as proxies for age, like eyeball size or the length of the cephalothorax. Comparisons of such easily measured biometrics with length-at-age will provide a test to determine whether these metrics are reliable proxies of size-at-age as proposed.

Development of more robust and consistent methods to embed and section the eyestalks would be useful to increase the overall efficiency of the process. We lost a fair number of samples during preparation, mounting, or sectioning and thus reduced the overall number of samples in our analysis by up to 50% in some cases in the initial phases of the study. This suggests that sufficient practice in embedding and sectioning krill will be necessary to minimize sample loss that could potentially bias ageing of samples if sample loss is greater in some samples (e.g., smaller krill) than others (larger krill). Unlike otoliths that have a distinctive primordium that provides a fixed and known initial starting point from which to count or measure increment widths, eyestalks do not have any fixed point to anchor measurements, making the sectioning critical to examining growth and estimating age. Additionally, physiological studies to determine the mechanisms that result in the formation and timing of annual bands is critical to understand whether growth rates can be inferred from the distance between annual bands, as has been inferred by Krafft et al. [39]. Long-term, multi-year laboratory studies of marked animals could provide information on the timing and retention of annual marks. The development and training of ageing technicians will require a community effort to exchange knowledge, but will also require a large number of known-age animals for distribution to different groups. Laboratories that can provide known-age animals to the community will be important in developing a competent and consistent ageing program within the Southern Ocean.

## Conclusion

The lack of methods to directly estimate the age of krill has been a major factor hindering the understanding of this species’ life history and has limited the development of predictive models for understanding the effects of climate change in the Southern Ocean on the biology of this critical species [2]. Recent breakthroughs in krill husbandry allowed us to raise krill of known age [48]. Using known-age krill from two different laboratories for reference, we have demonstrated that band counts from longitudinal sections of eyestalks reflect their absolute age. This is significant, as the use of known-age animals is considered the most rigorous age validation method because the absolute age of the animal is known without error [25].

The results of this work open the possibility of developing age-based assessment models in an increasingly important fishery. Development of age-based assessment methods would help to provide advice regarding stock structure, catch limits, and spatial management options as requested by CCAMLR [9]. Moreover, the Southern Ocean is undergoing major changes owing to climate change that have considerable impacts on the ecosystem [51]. These impacts include changes in sea ice [52], primary production [53], and ocean pH [54]. Because of the importance of krill to the Southern Ocean ecosystem, there is considerable interest in predicting the impacts of climate change on krill [23, 24, 55, 56]. Accurate predictions have been limited, in part because of the inability to quantitatively understand the plasticity of krill life history. We have also shown that formalin preservation of at least 23 years does not appreciably affect the ability to read eyestalk bands in these animals. Archived collections of krill exist for samples taken as far back as the 1920s, so the ability to age preserved krill provides the opportunity to retrospectively examine length-at-age in archived samples. It also provides the opportunity to compare length-at-age over time and across environments to examine changes in the Southern Ocean ecosystem, and will allow more robust predictions about the response of krill to the changes occurring in the Southern Ocean.

## Supporting information

S1 Fig Schematic diagram of the morphology of the eyestalk used for ageing. A schematic diagram showing the eyestalk from the anterior end (A) and top view (B), where the compound eye is visible filling most of the eyestalk mass. After preparing a longitudinal section in the clean structure, (C) shows the structure of the thin section showing the three main cuticle layers as follows starting from the outside: exocuticle (tan), endocuticle (red) and endocuticle (blue). All growth bands were observed and counted in the endocuticle (black bands).
(TIF)

S1 File Supporting data used in ageing known age and wild caught krill. This Microsoft Excel file contains the ageing data from wild caught Antarctic krill collected between 1992 and 2005 and preserved in formalin (summer) and krill collected in August 2015 (winter) and preserved in the Ethanol: water: glycerol preservative (Field Data). Data from known age krill used for age validation, and precision assessment of known age Antarctic krill are in Validation_data tab.
(ZIP)
