# Supplementary figures and images for: Validation of band counts in eyestalks for the determination of age of Antarctic krill, Euphausia superba
Source: PLoS One. 2017 Feb 22;12(2):e0171773. doi: 10.1371/journal.pone.0171773 (PMC5321404; doi:10.1371/journal.pone.0171773)

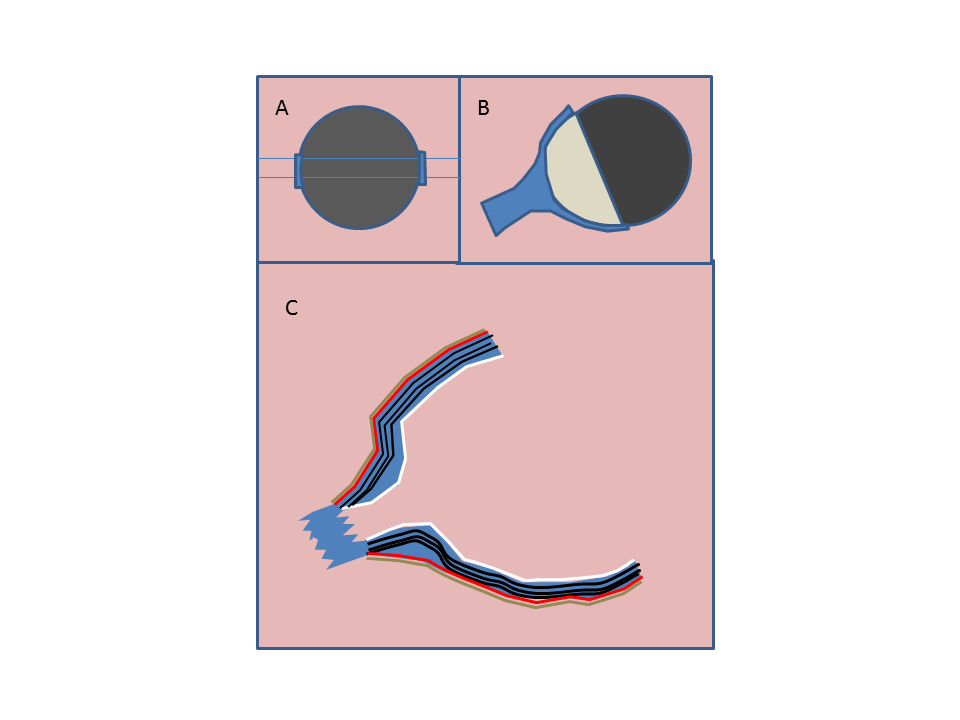

Supplement: S1 Fig — A schematic diagram showing the eyestalk from the anterior end (A) and top view (B), where the compound eye is visible filling most of the eyestalk mass. After preparing a longitudinal section in the clean structure, (C) shows the structure of the thin section showing the three main cuticle layers as follows starting from the outside: exocuticle (tan), endocuticle (red) and endocuticle (blue). All growth bands were observed and counted in the endocuticle (black bands). (TIF) [file pone.0171773.s001.tif]
